# Spatial maps and oscillations in the healthy hippocampus of Octodon degus, a natural model of sporadic Alzheimer’s disease

**DOI:** 10.1038/s41598-022-11153-4

**Published:** 2022-05-05

**Authors:** Matias Mugnaini, Diana Polania, Yannina Diaz, Marcelo Ezquer, Fernando Ezquer, Robert M. J. Deacon, Patricia Cogram, Emilio Kropff

**Affiliations:** 1 Leloir Institute—IIBBA, CONICET, Buenos Aires, Argentina; 2 Department of Ecological Sciences, Institute of Ecology and Biodiversity, Faculty of Sciences, Universidad de Chile, Santiago, Chile; 3 Centro de Medicina Regenerativa, Facultad de Medicina, Clínica Alemana-Universidad del Desarrollo, Santiago, Chile; 4 The Center for Neural Circuit Mapping, University of California, Irvine, Irvine, CA 92697 USA

**Keywords:** Neuroscience, Hippocampus, Spatial memory

## Abstract

The *Octodon degus* is a South American rodent that is receiving increased attention as a potential model of aging and sporadic late-onset Alzheimer’s disease (AD). Impairments in spatial memory tasks in *Octodon degus* have been reported in relation to either advanced AD-like disease or hippocampal lesion, opening the way to investigate how the function of hippocampal networks affects behavior across AD stages. However, no characterization of hippocampal electrophysiology exists in this species. Here we describe in young, healthy specimens the activity of neurons and local field potential rhythms during spatial navigation tasks with and without objects. Our findings show similarities between the *Octodon degus* and laboratory rodents. First, place cells with characteristics similar to those found in rats and mice exist in the CA1 subfield of the *Octodon degus*. Second, the introduction of objects elicits novelty-related exploration and an increase in activity of CA1 cells, with location specific and unspecific components. Third, oscillations of the local field potential are organized according to their spectral content into bands similar to those found in laboratory rodents. These results suggest a common framework of underlying mechanisms, opening the way to future studies of hippocampal dysfunction in this species associated to aging and disease.

## Introduction

Neural activity in the hippocampus of rodents has been studied for decades, especially following the seminal findings correlating spatial navigation with the activity of place cells^1^ and theta oscillations^2^ in the CA1 subfield of rats^3^. Place cells are pyramidal neurons that tune their activity to a given location in space, different across cells and environments^4^. While hippocampal lesions have been shown to disrupt performance in numerous spatial tasks^5,6^, evidence of a causal relationship between the firing of place cells and spatial orientation has recently begun to accumulate^7^. CA1 place cells have also been shown to respond to numerous variables other than spatial, in particular to the presence of objects^8,9^ or to their displacement^10^.

Theta oscillations and other rhythms recorded in the hippocampal local field potential (LFP), mainly classified according to their spectral content, are thought to reflect different levels of synchronization across neuronal populations^11,12^. The suppression of theta oscillations is associated with impairments in spatial memory tasks^13,14^. Features of theta (6 to 12 Hz), delta (1 to 5 Hz) and gamma (35 to 100 Hz) oscillations correlate with movement patters^15–18^. Gamma oscillations have been also related to dynamic routing of information in CA1^19^, while the much less studied beta band (15 to 30 Hz) has been associated to odor tasks and synchronization with odor-processing areas^20^. Another characteristic pattern of the CA1 LFP is represented by sharp wave ripple complexes, which are short isolated events during which high frequency ripples (120 to 200 Hz) coexist with low frequency irregular sharp waves^21^. They are thought to be associated to planning during the awake state or memory consolidation during sleep^22^.

Most of what we know about hippocampal spatial maps and LFP oscillations comes from the study of rats and mice. While this specialization has been a successful strategy for research groups to build a common knowledge base, the eventual irruption of studies on other species has allowed for conceptual breakthroughs. For example, bats have been found to exhibit spatial maps similar to those of rats, but no continuous theta oscillations, which allowed for testing of theories about the relationship between these phenomena^23^. Unconventional species also allow for the study of aspects of cognition related to their particular ethology, such as three-dimensional spatial maps discovered in flying bats^24^. This approach profits from the fact that the structure and function of the hippocampus have been well preserved across mammalian evolution^25,26^.

In this work we report for the first time hippocampal electrophysiology related to spatial navigation and spontaneous object exploration in the *Octodon degus* (degu), a natural animal model that exhibits features of sporadic late-onset Alzheimer disease (AD) and aging. The degu is a diurnal caviomorph rodent endemic to Chile. Previous studies have shown that some aged degus develop AD-like cognitive impairments and neuropathological features including beta-amyloid plaques, hyperphosphorylated tau neurofibrillary tangles and brain neuroinflammation, in conjunction with AD/aged-associated comorbidities such as macular degeneration, atherosclerosis, and type-2-diabetes^27–32^. Cognitive deficits in affected older degu are analogous to those in humans since they present a prodromal mild cognitive impairment-like stage, memory deficits, and impairments in species-typical behaviors, which may be comparable to impairments in activities of daily living in patients with AD^29,30,32^.

Aging and AD are associated with spatial memory impairments and with alterations in the encoding of information by the hippocampus. Studies of laboratory rodent models of AD have shown that spatial maps tend to deteriorate in correlation with the appearance of AD biomarkers and cognitive impairment^33–35^. In the context of translational research, mouse models have greatly improved our understanding of early-onset AD. However, the field lacks useful long-lived models of the more common sporadic late-onset AD and aging processes. The degu is a strong candidate model to study how sporadic AD affects function in the hippocampus and surrounding areas, the mechanisms behind impairments of the cognitive map and associated electrophysiological biomarkers. However, although the degu medial temporal networks are anatomically similar to those of laboratory rodents^36^, whether or not the similarity extends to electrophysiological features such as place maps and oscillations in the hippocampus is currently unknown. Here we aim to characterize neural activity in the dorsal CA1 subfield of young, healthy degus. This characterization sets the baseline for future work with aged degus in the study of the pathogenesis of late-onset AD.

## Results

### Spatial maps

**Figure 1:**
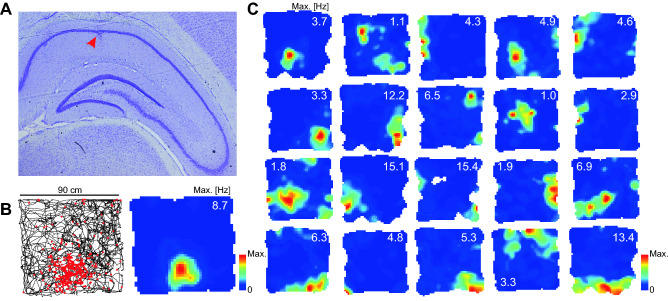
Place cells in the CA1 subfield of the degu hippocampus. (**A**) Representative example of cresyl violet stained histology showing the dorsal hippocampus and the recording site (red arrow). (**B**) Map of a principal cell with spatial field. Left: trajectory in a square environment (black) and positions where spikes were fired (red dots). Right: firing rate (color coded) version of the same map (maximum firing rate indicated). (**C**) Similar maps of all other recorded cells with significant spatial modulation.

No evidence exists to date on hippocampal place cells in the degu, although an anecdotal report exists in its close relative the chinchilla^37^. To understand if the firing properties of degu CA1 principal cells resemble those found in laboratory rodents, we recorded neural activity in five degus while they foraged freely in a 90 cm wide square environment and studied their spatial maps. We found that some of the cells recorded in each of our animals exhibited maps qualitatively similar to those of place cells (Fig. 1 and Supplementary Figs. S1, S2).

**Figure 2:**
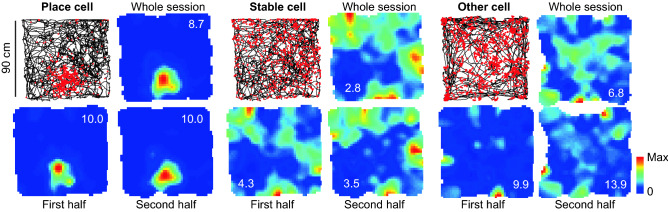
Spatial information and stability determine three cell categories. From left to right, an example of a place cell, a stable cell and a cell that is not informative or stable. For each cell, four maps are shown. Top: spike map (left) and rate map (right) for the data corresponding to the whole session. Bottom: rate maps for the first (left) and second (right) halves of the session.

**Figure 3:**
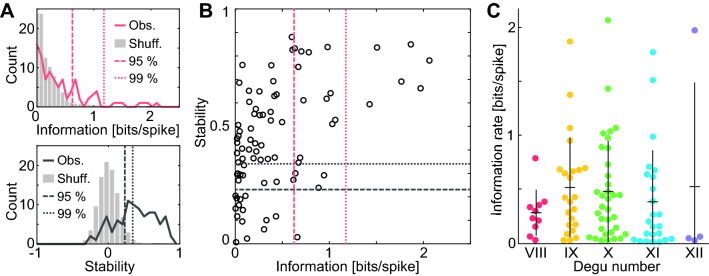
Statistics of spatial information rate and stability across cells and degus. (**A**) Spatial information rate (top) and stability (bottom) for the observed (color) and shuffled (grey; normalized by number of shuffles) distributions. Dashed lines indicate the 95th (information: 0.6 bits s^−1^, stability: 0.23) and 99th (information: 1.2 bits s^−1^, stability: 0.34) percentiles of the shuffled distribution. (**B**) Stability and information rate for individual cells (one circle per cell). Dashed lines as in (**A**). (**C**) Distribution of spatial information rate across degus.

To quantitatively characterize the spatial tuning of these maps we calculated their spatial information rate^38^. In addition, for each cell we calculated the spatial stability as the pixel-to-pixel correlation between the maps obtained for the first and second halves of the 20-min open field session. We observed that cells could be divided into three groups: (i) place cells with high information and stability, (ii) spatially stable cells with low spatial information content and (iii) spatially unstable cells (Fig. 2). To obtain an unbiased classification of cells according to spatial information and stability, we used thresholds based on percentiles of the distribution of these variables for the pool of maps obtained by shuffling the spike timestamps (1000 shuffles per cell; Fig. 3A,B; see Methods). For spatial information we found that 26 out of 95 cells (27%) exceeded the 95th percentile (0.6 bits s^−1^) of the shuffled distribution, while 7 (7%) exceeded the 99th percentile (1.2 bits s^−1^). These values were rather small compared to those obtained in laboratory rodents^39^. To understand if the population of informative cells was significantly larger than expected by chance, we compared the percentage of cells exceeding the 95th or 99th percentile with the outcome of a Bernoulli process with probability 0.05 or 0.01, respectively. We found that our dataset contained significantly more informative cells than expected by chance both with the 95th percentile (p: 10^−13^) and the 99^th^ percentile (p: 10^−5^) criteria.

In contrast with the rather low number of informative CA1 neurons, substantially more cells (67 out of 95 or 70%) exceeded the 95th percentile of stability (0.23), while 55 (58%) exceeded the 99th percentile (0.34). As before, both classification criteria resulted in groups of stable cells larger than expected by chance from the corresponding Bernoulli processes (p < 10^−100^ in both cases). Most spatially selective cells were also stable, as reflected by the fact that 21 cells (22%) had both spatial information and stability above the 95th percentile of the shuffled distribution, while this was true for 6 cells (6%) when using the 99th percentile thresholds. Cells with high spatial information were found in all studied animals (Fig. 3C; mean for cells above the 95th percentile threshold: 25%, min: 10%, max: 39%), and no significant differences were found between males and females regarding stability (Mann–Whitney test p: 0.06) or spatial information (Mann–Whitney test p: 0.19).

Put together, these results suggest that the degu CA1 contains place cells similar to those described in laboratory rodents, although in a smaller proportion, while many more principal cells exhibit spatially stable maps with lower information content.

### Object novelty

**Figure 4:**
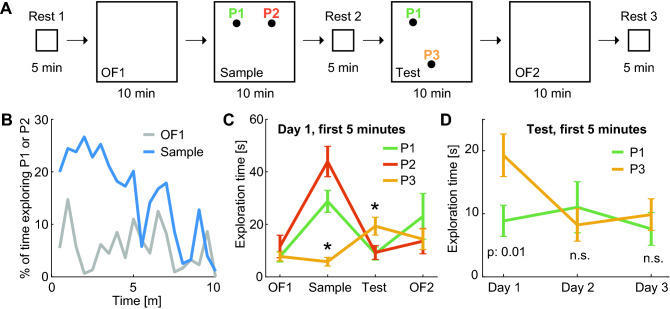
Degus preferentially explore objects introduced in novel positions. (**A**) Diagram of consecutive sessions in the novel object location experiment. Identical objects are introduced in a familiar environment in positions P1 and P2 in the Sample session and in positions P1 and P3 in the Test session. (**B**) Percentage of consecutive 30 s windows exploring positions P1 or P2 during the OF1 (grey) and Sample (blue) sessions (Wilcoxon test for difference during first 5 m z: 3.7, p: 2 10^–4^). (**C**) Overall exploration time around P1, P2 and P3 (mean ± s.e.m.; color code as in **A**) during the first 5 min of each session in day 1. Asterisks indicate position explored significantly more or less than others (p < 0.05). (**D**) As in (**C**), overall exploration time of positions P1 and P3 during the first 5 min of the Test session across non-consecutive days. P3 was more explored than P1 on day 1 (p: 0.01) but not on the following days.

In the wild, degus exhibit scatter hoarding, which involves placing small caches of food in hidden places, generally underground. To assess their interest in objects and object location in classical laboratory settings, as well as potential neural correlates in the hippocampus, we trained three subjects in a short-term memory variant of the novel object location task (Fig. 4A). Each degu initially explored a familiar open field (90 cm wide square; OF1). Then it was exposed to the same environment with two objects in positions P1 and P2 (Sample). After a 5 min rest, it was put into the same environment, where one of the objects had been displaced from P2 to P3 (Test). Laboratory rodents typically exhibit a spontaneous preference for the exploration of the displaced object during this session for a range of variations of the task^40,41^. Finally, the subject explored the same environment without objects (OF2). The experiment was repeated in 3 non-consecutive days for each degu.

To quantify object preference, we evaluated the time spent in a circle of 10 cm around P1, P2 and P3 during consecutive 30 s windows. We found that during the first 5 min of the Sample session on day 1, the exploration of the newly introduced objects (pooled for all animals) was higher than the exploration of the same empty locations during the previous OF1 session (Fig. 4B; medians over pooled non-consecutive 30 s windows: 22% vs. 5.6% of time; Wilcoxon test z: 3.7, p: 2 10^–4^). However, in agreement with the progressive loss of interest in newly introduced objects exhibited by laboratory rodents^40^, the difference was not significant for the second halves of the same 10 min sessions (Wilcoxon test z: 1.5, p: 0.15), which led us to consider only the first 5 min of each session for all further comparisons. We next compared more generally the exploration of P1, P2 and P3 across sessions of day 1 (Fig. 4C). We found differences in exploration time related to position during the Sample (Kruskal–Wallis test H(2): 35.5, p: 10^–7^) and Test (H(2): 8.1 p: 0.017) sessions, but not during OF1 (H(2): 0.12, p: 0.9) or OF2 (H(2): 0.7, p: 0.7). Post-hoc Tukey–Kramer multiple comparison analyses indicated that the only significant differences were between P3 and the other two positions. During the Sample session, when objects were placed in P1 and P2, P3 was significantly less explored (p < 0.01). During the Test session, when P3 contained the displaced object, it was significantly more explored (p < 0.05) than P1 (object in a familiar location) or P2 (no object). This novelty effect related to exploration of the displaced object was not observed in subsequent repetitions of the experiment. P3 was more explored than P1 during the Test session on day 1 (Wilcoxon test z: 2.45, p: 0.014), but not on the following days (Fig. 4D; p > 0.15). These results suggest that, as observed in laboratory rodents, longer object exploration times in degus are associated to object or object-location novelty, and that differences in the exploration time of objects vanish as objects or tasks become familiar.

**Figure 5:**
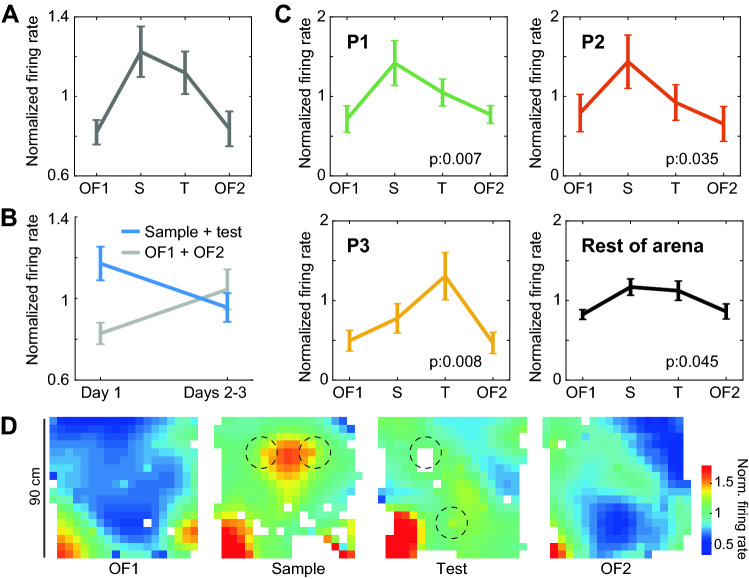
Cells exhibit a combination of global and local response to the novel placement of objects. (**A**) Distribution across sessions of the mean normalized firing rate for 18 neurons recorded on day 1 (mean ± s.e.m.; Kruskal–Wallis test H(3): 9.6, p: 0.023). (**B**) As (**A**) but pooling sessions with (blue) and without (grey) objects across days. A significant difference was found in Day 1 (Wilcoxon test z: 2.96, p: 0.003). (**C**) Similar plots for circles 10 cm in radius around each position (indicated) or for the rest of the maze across sessions of day 1. Significance of a Kruskal–Wallis test comparing sessions indicated (P1 p: 0.007, P2 p: 0.035, P3 p: 0.008, Rest of maze p: 0.045). (**D**) Mean normalized maps for all sessions on day 1. Circles 10 cm in radius used for analyses are indicated (dashed lines). Note that smoothing in these maps has the purpose of improving visualization, and does not affect results in (**A**–**C**), where no binning or smoothing was used.

We next examined the activity of CA1 principal cells recorded in this experiment (day 1: 17 cells, day 2: 8 cells, day 3: 9 cells). A visual examination revealed great variability in individual neural responses (Supplementary Figs. S3, S4), which led us to look for effects at the population level. To compare changes in firing rate across sessions with equal weight for all neurons, the activity of each neuron was normalized by its firing rate averaged over all 4 sessions of the day. We observed that on day 1 the normalized firing rate increased, on average, during the Sample and Test sessions, compared to OF1 and OF2 (Fig. 5A). The effect of session type was significant (Kruskal–Wallis test H(3): 9.6, p: 0.023), and a Tukey–Kramer post-hoc analysis indicated that this was only due to a rather borderline difference between OF1 (median: 0.89) and Sample (median: 1.11) sessions (p: 0.038). To understand if the significance of these results was close to borderline because of the low number of neurons available, we pooled sessions with (Sample + Test) and without (OF1 + OF2) objects, doubling the number of observations in each group, and obtained a more significant difference in firing rate between these two groups (Fig. 5B; objects median: 1.09; no-objects median: 0.9; Wilcoxon test z: 2.96, p: 0.003). We next aimed to understand if this difference was also present in the following days. We pooled data from days 2 and 3 to get a similar number of neurons (17), but did not observe a difference between sessions with and without objects (objects median: 0.99; no-objects median: 1.01; Wilcoxon test z: − 0.31, p: 0.75). These results are in line with observations in rats, suggesting that an overall increase in firing rate of CA1 principal cells is associated to novelty in object identity or location^10^.

Next, we asked if beyond the global increase in activity there was a specific increase in firing rate around objects. We computed for each neuron the mean normalized firing rate in circles of 10 cm around P1, P2 and P3, as well as in the area outside the union of these circles. For each of these areas, we plotted the distribution of normalized firing rate across cells and sessions (Fig. 5C). We observed that in P1, P2 and P3 the activity peaked on the session during which an object was first presented. In particular, neurons fired more during the Sample session than during OF1 in locations P1 and P2 (right-tail Wilcoxon tests: z: 2.46, p: 0.007 and z: 1.8, p: 0.035, respectively), and more during the Test session than during OF1 in location P3 (right-tail Wilcoxon test: z: 2.41, p: 0.008). The firing rate in the area outside of the circles varied across sessions in a way similar to the one observed for the global firing rate (Kruskal–Wallis test: H(3): 8.04, p: 0.045). This indicates a global increase in firing rate during object relative to no-object sessions, even in positions away from objects. To visualize this combination of global and local variations in firing rate, maps for all neurons on day 1 (normalized by global firing rate) were averaged for each session (Fig. 5D; 5 cm bins were used to improve visualization due to poor coverage away from objects during object sessions). In this visualization, the area between P1 and P2 during the Sample session appears as the location with the strongest local increase due to the introduction of objects. This qualitative observation needs to be tested in future studies involving higher numbers of neurons.

Put together, these results suggest that in the degu novel objects or novel object locations induce a combination of a location-unspecific and a location-specific increase in firing rate of CA1 principal cells, which declines with familiarity. Similarly, preference for displaced objects also declines with familiarity, in line with observations in laboratory rodents.

### Local field potential oscillations

**Figure 6:**
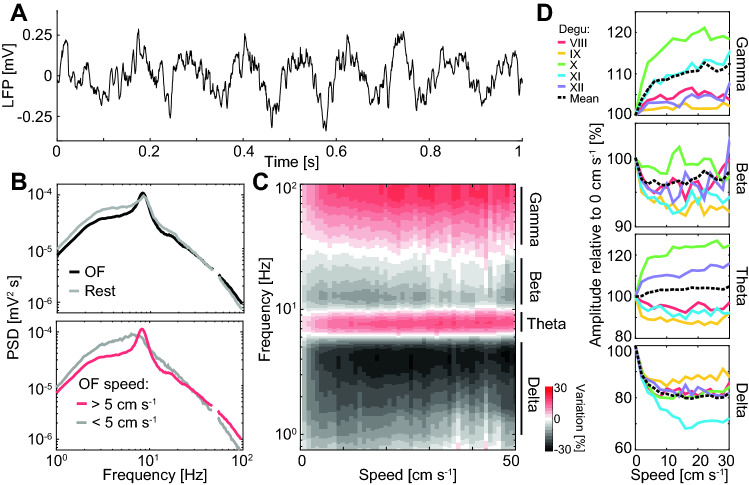
Relation to motion determines frequency bands in the local field potential. (**A**) Representative example of 1 s of local field potential during navigation in the open field. (**B**) Median power spectral density for consecutive 2 s windows. Top: windows in rest (grey) vs open field (black) sessions. Bottom: windows of slow (grey) vs fast (red) mean running speed during open field sessions (cutoff: 5 cm s^−1^). Window around 50 Hz (power line frequency) is removed to improve visualization. (**C**) Mean variation in wavelet transform amplitude relative to the value at 0 cm s^−1^ for different frequencies and running speeds. Regions of positive and negative relationship with running speed determine frequency bands (indicated) that mirror those found in laboratory rodents. (**D**) Mean variation in Hilbert transform amplitude as a function of speed for the frequency bands identified in (**C**) (from top to bottom: gamma, beta, theta and delta) for the 5 degus recorded in open field experiments (color coded) and overall mean (dashed line).

The LFP that we recorded in the degu CA1 exhibited oscillations that recall those found in laboratory rodents (Fig. 6A). A clear theta rhythm was directly observable in raw LFP plots. Given the variability in LFP features, further characterization, unless specified, was done only on open field sessions and tetrodes from which cells were recorded (channels per animal min: 36, max: 120, mean: 76.8). The median power spectral density for the pool of all selected tetrodes exhibited a peak at 8.2 Hz with half-decay width at 6.4 Hz and 9.7 Hz (Fig. 6B; the median was used instead of the mean to give less relative weight to the channels with highest amplitude oscillations, given the strong variability; here and in some subsequent plots small windows around the power line frequency of 50 Hz and its harmonics are removed to improve visualization). In power spectral density plots for rest sessions there was a reduction of the theta peak accompanied by an increase in power at lower frequencies, consistent with properties of delta band oscillations observed in laboratory rodents^18^. To understand if this could be related to mobility/immobility states, we divided the open field data into consecutive 2 s windows and obtained for each window the mean speed and the power spectral density. When comparing slow and fast windows (cutoff speed: 5 cm s^−1^), the decrease in theta and increase in delta associated to immobility were even more marked then in the open field vs rest session comparison (Fig. 6B).

These findings led us to ask more generally about running-related changes in power across a wide spectrum of frequencies. To account for instantaneous variations, we examined the wavelet amplitude of the LFP for frequencies ranging from 1 to 100 Hz. For each channel and frequency, we calculated the percentage of variation in wavelet amplitude at a given instantaneous speed relative to the amplitude at 0 cm s^−1^, and averaged across channels (Fig. 6C). Four different regimes were observed, which allowed us to define the frequency bands used in the rest of the manuscript. The delta band (1 to 5 Hz) had the strongest relative decrease in wavelet amplitude associated to speed (max: 28%), while the theta band (6 to 11 Hz) had an increase (max: 16%), consistent with previous observations using power spectral density plots. Faster oscillations could be divided into beta and gamma bands. The beta band was characterized by a reduction of up to 12% in average wavelet amplitude due to running if defined from 10 to 30 Hz. However, we used a more conservative definition (from 20 to 30 Hz) to avoid possible confusions with the first theta harmonic, which was particularly strong in some channels. With this definition, the maximum reduction in amplitude was 9%. The gamma band (35 to 100 Hz) was characterized by a relative increase in average wavelet amplitude due to running of up to 20%. Bands defined in this way are remarkably similar to those described in laboratory rodents, with similar speed-related responses^12,18,42^.

Next, we asked if these mean trends were preserved across individuals. To study the relationship between speed and instantaneous variations in amplitude of whole frequency bands, we used the Hilbert transform of the band-pass filtered LFP. As in the previous plot, for each individual degu and for different speeds we plotted the mean band amplitude as a percentage relative to the corresponding value at 0 cm s^−1^. To quantify variations associated with running, we performed Wilcoxon signed ranked tests for the data from 10 to 30 cm s^−1^. The decrease in delta and beta amplitude due to running speed, as well as the increase in gamma amplitude, were significant in all animals (p < 0.01). In contrast, instantaneous variations in theta amplitude due to running speed presented high variability across animals. Two subjects presented a significant increase and two a significant decrease, while variations in one animal (degu XI) were not significant (p: 0.3).

Put together, these results indicate that the LFP of degus is divided roughly into the same bands as those of rats and mice. Power in most bands presents a relationship with motion similar to that of laboratory rodents, although the average increase in theta power with running speed is not robust across subjects.

### Rhythmic spiking

**Figure 7:**
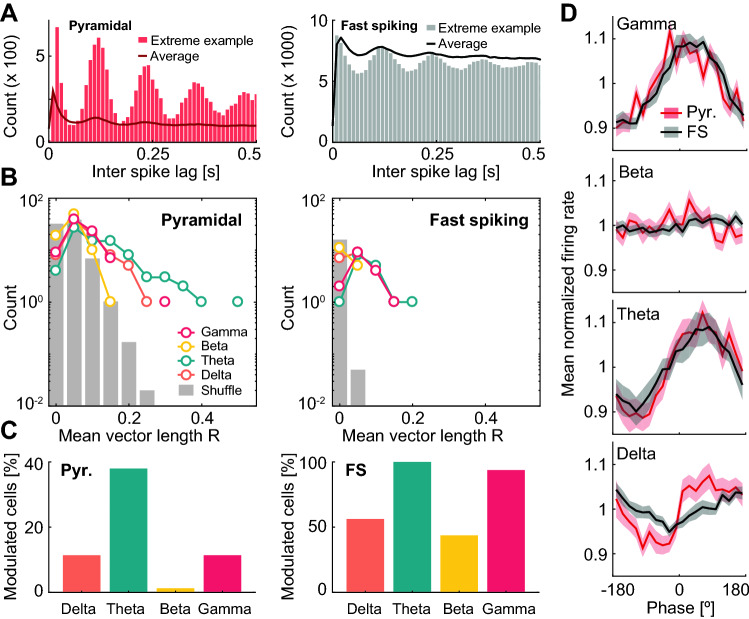
Frequency bands modulate the spiking activity of CA1 neurons. (**A**) Spike-lag histograms for the putative pyramidal (left) and fast spiking (right) cells with strongest theta modulation (bars) together with the population mean for each cell type (line). (**B**) Distribution of mean vector length R for putative pyramidal (left) and fast spiking (right) cells (frequency bands color coded). Grey bars: distribution of the pool of 100 shuffles in spike timestamps per cell. (**C**) Percentage of putative pyramidal (left) and fast spiking (right) cells with significant modulation for each frequency band (same color code). (**D**) Normalized firing rate as a function of oscillation phase for putative pyramidal (red) and fast spiking (black) cells for different frequency bands (from top to bottom: gamma, beta, theta, delta; mean ± s.e.m.).

In order to study the rhythmic spiking of recorded cells in relation to LFP oscillations, we first observed spike lag histograms^43^. To differentiate between putative principal and fast spiking cells, we used the mean firing rate as a criterion with a cutoff value of 7 Hz. Modulation of firing by theta rhythm was evident in some examples of both cell types (Fig. 7A). To quantify it, we obtained for each cell and each frequency band the mean vector length (R) of the spiking phase (obtained from the Hilbert transform of the LFP). We compared these R values with the distribution obtained from pooling 100 shuffles of the spike timestamps for each cell (Fig. 7B), and calculated the number of cells classified as modulated by each frequency band (Fig. 7C). In the pyramidal cell population, modulation by theta was by far the strongest (38% of cells), followed by gamma (11%) and delta (11%). Only 3 cells (4%) were modulated by beta. Individual putative fast spiking cells exhibited mean values of R lower than those of putative pyramidal cells for all four frequency bands, but shuffled R values were much lower due to the higher number of spikes per cell. As a consequence, almost all fast spiking cells were classified as significantly modulated by theta (100%) and gamma (94%), while around half of the population was significantly modulated by delta (56%) and beta (44%).

To understand the mean effect of these modulations at the population level, we next plotted for each band the firing rate (normalized, as previously, by mean firing rate, to give equal weight to every cell) as a function of the oscillation phase (Fig. 7D). In all cases the modulation was similar for putative pyramidal and fast spiking cells, mostly involving variations of around 10% in the normalized mean firing rate. This was true for all bands with the exception of the beta band, where both cell populations exhibited marginal variations suggesting a modulation of the activity of cells that, if present, was not coordinated across the population. Oscillations of the population normalized firing rate tended to be in phase with theta (circular mean pyramidal cells: 66°, fast spiking cells: 60°) and gamma (circular mean pyramidal cells: 17°, fast spiking cells: 33°) rhythms, and close to out of phase for the delta band (circular mean pyramidal cells: 102°, fast spiking cells: 160°).

### Sharp-wave ripples

**Figure 8:**
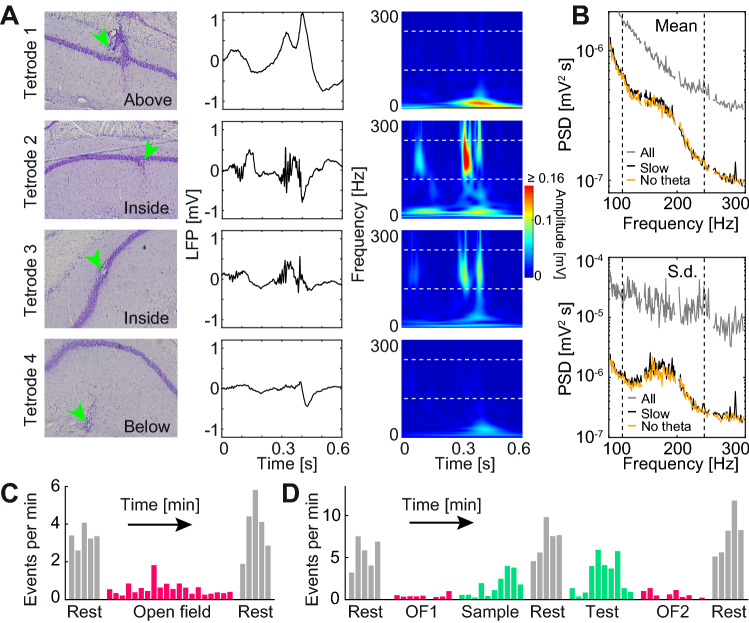
Sharp wave ripple complexes in degus. (**A**) Representative example of a sharp wave ripple complex (SWR) recorded simultaneously at four different locations (one tetrode per row; from top to bottom: above, inside, inside and below the CA1 pyramidal cell layer). Left: histology of degu XII showing the position of each tetrode (green arrow). Center: local field potential during a 0.6 s window (the same for all tetrodes). Right: corresponding spectrogram obtained with the wavelet transform. White dashed lines: 120 and 240 Hz. (**B**) Mean (top) and standard deviation (bottom) of power spectral density for the distribution of 2 s windows corresponding to all selected channels in open field recordings (grey), for the selection of windows with low mean running speed (6% of windows; black; cutoff: 1 cm s^−1^) and for the selection of windows within prolonged periods of low theta amplitude (3%; orange). Dashed lines: 120 and 240 Hz. (**C**) Mean count of SWR events for consecutive 1 m windows (1 bar per window) during consecutive rest (grey) and open field (red) sessions. (**D**) As (**C**) but for rest (grey), open field (grey) and object (green) sessions in the novel location experiment. More events occurred during rest (mean: 6.7 min^−1^) than during sessions with objects (mean: 2.3 min^−1^) or open field sessions (mean: 0.5 min^−1^) (Kruskal–Wallis test H(2): 37.3, p: 10^–8^; Tukey–Kramer post-hoc analysis indicated that all 3 session types where significantly different from each other, p < 0.01).

To understand if sharp-wave ripple complexes (SWRs) are present in the degu CA1, we analyzed the wavelet transform of the LFP using frequencies up to 300 Hz. We observed events resembling SWRs reported in laboratory rodents, during which ripples in the pyramidal cell layer co-occur with sharp-waves above and below it (Fig. 8A). A peak in the mean and standard deviation of the power spectral density in the range of 120–240 Hz was evident for the pool of 2 s windows of LFP characterized by an average running speed below 1 cm s^−1^, but absent when windows of all speeds were included, consistent with the association of SWRs with waking immobility (Fig. 8B). Another salient feature of SWRs in awake animals is that they tend to occur during episodes of prolonged absence of theta oscillations^44^. To identify such episodes, we set the condition that the instantaneous wavelet amplitude at 8 Hz did not exceed in more than 40% the instantaneous wavelet amplitude at 6 Hz, both signals smoothed with a 15 s moving average window. This criterion, as the low speed one, led to the emergence of a SWR associated peak in the spectral density (Fig. 8B). We next asked if different types of session would be associated with a different number of SWRs. Individual events were defined as peaks above 5 standard deviations in the amplitude of the whole-band (120 to 240 Hz) Hilbert transform of the LFP, occurring during prolonged periods of low theta amplitude (same criterion as in Fig. 8B). Defining SWR events using the low-theta criterion, which could be applied also to rest sessions were no tracking was available, we analyzed open field experiments (5 animals). We observed more events during rest sessions than during consecutive open field sessions (Fig. 8c; means of pooled non-overlapping 1-min windows: 3.6 vs 0.6 events per minute, Wilcoxon test z: 4.4, p: 10^–5^). Type of session also modulated the frequency of occurrence of SWR events in our experiment with objects (Fig. 8D; 3 animals). More events occurred during rest (mean: 6.7 min^−1^) than during sessions with objects (mean: 2.3 min^−1^) or open field sessions (mean: 0.5 min^−1^) (Kruskal–Wallis test H(2): 37.3, p: 10^–8^; Tukey–Kramer post-hoc analysis indicated that all 3 session types where significantly different from each other, p < 0.01).

Put together, these results suggest that SWRs occur in the hippocampus of the awake degu in a behavior-dependent manner, with characteristics similar to those observed in laboratory rodents.

## Discussion

Our characterization of CA1 electrophysiology in young, healthy degus points to many similarities with well-studied properties of hippocampal function in laboratory rodents. These similarities include the response of hippocampal neurons to the animal’s spatial location, with and without objects, as well as the spectral organization of the local field potential into well-defined bands and their relationship to motion. Within this framework, some specific properties in which degus seem to deviate from what has been observed in other species were also found, such as an unexpected variability in the relationship between theta amplitude and running speed. Despite these isolated differences, our results open the way to the study of CA1 electrophysiology during naturally occurring late-onset Alzheimer’s (AD) -like disease. This approach will benefit on one hand from the wealth of accumulated knowledge on the function and mechanisms of rodent hippocampal circuits, and on the other hand from the fact that, unlike in most AD models, the disease in degus presents many features that relate it to the most prevalent sporadic form of AD in humans.

Our results suggest that degus have place cells with a spatial tuning similar to what has been found in laboratory rodents and other mammals, although neurons with strong tuning appear less frequently. Classification methods have evolved throughout the years, which make comparisons hard in general terms. We have adopted the classification based on shuffled percentiles of the spatial information, due to its unbiased nature. While our results show that 27% of cells in the degu CA1 have spatially tuned maps, studies applying similar classification criteria suggest that this proportion is 60% in rats^45^, 50% in mice^34^ and 36% in bats^23^. However, to contextualize this comparison it should be noted that non-place cells have been shown to be able to effectively contribute to the spatial population coding, so that position can be in some cases decoded from non-spatially tuned cells alone^46^. In this sense, although high spatial tuning in the degu seems to be somewhat rare, spatially stable cells were found in greater numbers. Stability statistics is not often reported, which makes cross-species comparisons difficult. Distributions of spatial stability have been directly compared for rats and mice running in a linear track, and no significant difference between medians was found^39^ (mice: 0.59, rats: 0.67). Although in a different setup (open field), the median stability of 0.37 that we found for degus looks substantially smaller, which contributes to an overall picture of the degu CA1 as capable of coding for space through sparse representations but somewhat less specialized in it.

Although objects are a widespread tool to study memory and response to novelty in the rodent hippocampus, the study of neural correlates of the interaction with objects has only recently captured widespread attention^8^. Our results show that degus exhibit a preference to explore novel objects or familiar objects at a novel location. This preference adapts with repetition, as is common in similar studies with laboratory rodents. CA1 neurons respond to the introduction of objects with a location unspecific increase in firing rate similar to what has been described in rats^10^. In addition, their firing around locations where objects are placed peaks on the first presentation of the object. Both responses (location-specific and the location-unspecific) adapt with repetition. These results obtained with very few subjects suggest that experiments with objects are an attractive way to probe hippocampal activity in degu, especially relevant for future assessments of the effects of age and disease. In addition, efforts should be directed in the future to investigate other behaviors related to daily living, such as burrowing or nest building, that are among the earliest indicators of AD onset.

Clear cut local field potential bands are perhaps the feature of degu electrophysiology that most closely resembles that of laboratory rodents. The classical delta, theta, beta and gamma bands could be identified by noticing that cutoff frequencies existed where the relationship between LFP amplitude and running speed shifted its sign. These cutoff frequencies were extremely similar to those described in rats and mice, and the relationship between the amplitude of bands and running speed followed similar trends. In contrast, the finding that the relationship between theta power and running speed is variable across subjects is unexpected. Although it is hard to assess from reported studies the degree to which variability across subjects exists also in laboratory rodents, it is generally assumed to be much lower. Future efforts to clarify this could include a standardization of recording sites (for example using silicon probes) and behavior (for example using a running wheel).

Sharp wave ripple events are present in degus as in laboratory rodents, with a similar spectral content^21,39^. Our results point to the possibility that a common mechanism underlies their generation across these species, triggered especially during awake immobility and in the presence of objects. This is particularly important in the view that a decay in the abundance of sharp wave ripples has been pointed out as a potential early electrophysiological biomarker of AD onset^47^.

The understanding of the basic characteristics of the electrophysiology of the degu CA1 is a necessary first step towards describing the effects on neural coding of the sporadic late-onset AD-like pathology spontaneously exhibited by a fraction of aged subjects. The finding of a general framework of similarity with the much better understood electrophysiology of laboratory rodents paves the way toward utilizing this model in the future for basic and applied research related to the mechanisms behind hippocampal dysfunction in AD and aging.

## Materials and methods

Ethical approval for this project was given by the Institute of Ecology and Biodiversity Ethical Committee, University of Chile. Experiments were performed in this Institute in accordance with the requirements of the UK Scientific Procedures Act (1986). All animal experiments comply with the Arrive guidelines and the National Institutes of Health guide for the care and use of Laboratory animals (1978).

### Animals

Five young-adult (9–20 months old, mean ad libitum weight = 250 g) degus, 2 male (VIII and XI) and 3 female (IX, X and XII) from the outbred colony at the Institute of Ecology and Biodiversity (IEB, Faculty of science University of Chile, Chile) were used in this work. The degus were housed in standard metal cages, 50 × 40 × 35 cm, with a layer of wood shavings as bedding and containing a small metallic box (25 × 15 × 10 cm with a single entrance) under a controlled photoperiod (7 am–7 pm) and temperature (23 °C). Water and food were provided ad libitum. The degus were fed a commercial rodent diet (Prolab RMH 3000 laboratory diet, USA).

No statistical determination of sample size was used, as it is hard to estimate the number of neurons and the type of hippocampal maps obtained from any given animal in a species that has not been studied before. Statistical analyses were performed a posteriori using all available data.

### Surgery

Degus were chronically implanted with a four-tetrode microdrive, which was originally developed for mice (Versadrive-4, Neuralynx, USA). Tetrodes (~ 25-μm in diameter) were constructed from four strands of tungsten wire (99.95% tungsten, 12.5 μm in diameter, California Fine Wire Company, USA), bound together by twisting and melting their insulation with hot air (approximately 110 °C). Five tetrodes were loaded and glued into a nested assembly of stainless-steel tubes and mounted into the microdrive (total weight around 2.5 g). The tetrodes exited the microdrive through a guide cannula in an approximately rectangular arrangement (approximate spacing 400-μm) and every tetrode could be moved independently via a drive screw (160 μm per turn). Each tetrode was cut flat and its tip was gold-plated to reduce the 1 kHz impedance of individual wires to 0.2–0.4 MΩ. The surgery was conducted under sevoflurane anesthesia administered in oxygen (> 90%) as a carrier gas (1 l m^−1^ flow), in a concentration constantly regulated according to breathing patterns. A circular opening (approximately 1.5 mm in diameter) was made in the skull above the right dorsal hippocampus. The center of the craniotomy was − 3.2 mm lateral to the midline and − 4.3 mm posterior to bregma. These coordinates were adapted from a previous description of the degu brain^48^. After the removal of the dura, the microdrives were lowered 1 mm relative to the brain surface. Next, 7 jewler’s screws were inserted in holes drilled in the skull around the craniotomy, which was filled with a biocompatible transparent gel Neuraseal, obtained by combining 0.5% sodium alginate and 10% calcium chloride, both previously dissolved in distilled water and stored separately. The skull, the screws and the base of the microdrives were then covered with dental cement (auto-cristal acrylic, DentalLab). After surgery animals were left to recover for 5 days.

### Lowering of tetrodes

During a period of 1 week after surgery recovery, the tetrodes were progressively lowered toward the CA1 pyramidal layer. One of the tetrodes served as a reference and was left in an electrically-quiet zone (at brain surface, above the hippocampus). This reference tetrode was used for differential recordings. The remaining four tetrodes served as recording probes. Sharp waves and ripples were the main guides to position the electrodes on the pyramidal cell layer.

### Behavior

All experiments were conducted in the same 90 cm wide square arena, with 35 cm high wooden walls. The arena floor and walls were colored black to avoid LED reflections. A white cue card (20 × 30 cm) was placed in the west wall to facilitate orientation. No curtains were used and objects in the laboratory gave additional distal cues.

Open field experiments consisted of three consecutive sessions (5 min rest, 20 min open field foraging and 5 min rest) during which neural activity was recorded using neural loggers (Deuteron Technologies, Israel). During open field sessions degus were filmed from above using a camera (Basler, Exton, PA; sampling rate: 50 Hz) placed on the lab ceiling and the positions of a green and a red LED attached to the loggers tracked using the Ethovision software (Noldus, Wageningen, the Netherlands). Speed was obtained as the distance between tracked positions over a temporal window of 200 ms, divided by 200 ms. During all open field sessions sunflower seeds were sporadically thrown into the arena at random locations to encourage foraging. Rest sessions took place in a resting box (30 cm wide square) outside the range of the camera. Open field experiments were repeated an average of 5 times during non-consecutive days (range: 2 to 8). After every open field session, the surfaces of the arena were cleaned with 70% ethanol.

Novel object location experiments consisted of 7 consecutive sessions (5 min rest, 10 min open field, 10 min sample, 5 min rest, 10 min test, 10 min open field, 5 min rest). Rest and open field sessions were similar to the ones previously described. Sample and test sessions had the addition of two objects in positions P1 and either P2 (sample) or P3 (test) (see diagram in Fig. 4a). Three groups of identical objects were used, one in each repetition of the experiment for each subject (48 h separation between repetitions), in a counterbalanced manner. Groups of identical objects were: (a) 20 cm high chess rooks, (b) 25 cm high glass bottles filled with sand and (c) 20 cm high cylindrical trash bins. Sunflower seeds were not administered during object sessions to favor interest in the objects.

### Data collection

Electrophysiological signals were acquired at a sampling rate of 32 kHz, using a bandpass filter of 1–7000 Hz prior to digitalization. Data processing was conducted with ad hoc MATLAB (MathWorks, Natick, MA) scripts, as well as all subsequent analyses. For LFP analyses, signals were low-pass filtered at 1 kHz (4th order Butterworth filter) and downsampled at 2 kHz to make them less computationally heavy. For spike sorting, the same signals were band-pass filtered between 300 Hz and 7 kHz (4th order Butterworth filter). Peaks exceeding 40 μV and separated by at least 0.5 ms were identified and their waveforms in a window ranging from 3 samples before to 30 samples after the peak were saved for all channels of the tetrode.

### Spike sorting

Events corresponding to potential neural spikes were manually clustered offline. Multiple low dimensional projections were simultaneously used to identify well separated clusters: the peak-to-peak voltage in each channel, the voltage at user-defined time points of particular channels and two dimensional tSNE reductions with default parameters (MATLAB function *tsne*). Autocorrelation and crosscorrelations were used as additional separation criteria.

### Spatial rate maps

Rate maps that showing firing rate as a function of location were obtained by spatially smoothing (1.6 bin standard deviation 2D Gaussian filter) the maps obtained as the ratio between spike count and time spent on a 2.5 cm side square bin grid covering all the arena. For visualization of average population activity in the novel location task (Fig. 5), 5 cm bins were used due to low coverage in some bins away from objects.

Given a spatial map with mean firing rate λ and a value λ_i_ for each of its N bins, information rate^38^ was computed as.

$$Information= \sum_{i=1}^{N}{p}_{i}\frac{{\lambda }_{i}}{\lambda } {log}_{2}\left(\frac{{\lambda }_{i}}{\lambda }\right),$$

where p_i_ is the occupancy probability of bin i. Stability was computed as the Pearson correlation between the maps obtained from the first and second halves of the session.

### Shuffling

Chance-level statistics was constructed for a given variable W through a shuffling procedure. At each repetition, the entire sequence of spikes fired by the cell was time-shifted along the animal’s path by a random interval between 30 s and the total trial length minus 30 s, with the end of the trial wrapped to the beginning. The shuffled instance of the variable W was then calculated using the shifted spikes, and the collection of 100 repetitions for each cell composed the chance-level statistics. For classification purposes, all shuffled data of the corresponding score was pooled together and the 95^th^ and 99^th^ percentile of the distribution were used as classification criteria.

### Significance of cell population number

Cell populations defined by a Kth percentile of the shuffled distribution criterion were compared with the expected outcome of a Bernoulli process with probability of success (1-k), where k = K/100. If success is equivalent to a cell passing the criterion by chance, the probability of obtaining a subpopulation of size S within a population of size N is

$$P\left(S\right)=\left(\begin{array}{c}N\\ S\end{array}\right){{(1-k)}^{S}k}^{N-S}$$

and the right tail p-value associated to S is

$$p=\sum_{l=S}^{N}P\left(S\right).$$

### Spectral analyses

To reduce variability, analyses of local field potential (LFP) included only channels from tetrodes that had cells. In an initial, unbiased approach, the power spectral density was analyzed. Each time series was divided into consecutive 2 s windows and the power spectral density for each window was obtained using the MATLAB function *periodogram()*. Different sets of windows were then selected and averaged according to the criteria of each analysis.

To study instantaneous variations in LFP and its relation with running speed for an unbiased range of frequencies, the Morlet wavelet transform was applied, giving as a result an instantaneous amplitude and phase for each frequency. To study the instantaneous behavior of specific frequency bands, the LFP was band-pass filtered within the limits of the band and the Hilbert transform was applied, giving as a result an instantaneous amplitude and phase representing the whole band.

To study sharp wave ripple events, episodes of prolonged low theta amplitude were defined. The criterion to define these episodes cannot be based on the instantaneous theta amplitude because the sharp wave often has a spectral content that overlaps with the theta band. Instead, the criterion was based on the ratio of wavelet amplitudes at 8 and 6 Hz, both smoothed using a 15 s moving window. This ensured that around the event theta amplitude was low even if the sharp wave itself contained frequencies within the theta band^44^. Events were defined as peaks in amplitude of the band-passed LFP (120 to 240 Hz) that exceeded 5 standard deviations, with a minimum spacing between events of 0.5 s.

### Histology

Electrodes were not moved after the final recording session. Subjects underwent inhalatory sevoflurane anesthesia and were perfused intracardially with 100 ml of washing solution (0.4% glucose, 0.8% saccharose and 0.8% NaCl) and then with 100 ml of 4% paraformaldehyde. The brains were extracted and stored in 4% paraformaldehyde, and frozen coronal sections (40 um) were cut. All sections were mounted on glass slides and stained with cresyl violet. With the use of a light microscope, equipped with a digital camera, the positions of the recording electrodes were registered in relation to relevant borders between subfields.

## Supplementary Information

Supplementary Information.

## Data Availability

The datasets generated during the current study are publicly available in https://github.com/cocogon/Degus.
