# Supplementary material for: Spatial maps and oscillations in the healthy hippocampus of Octodon degus, a natural model of sporadic Alzheimer’s disease
Source: Sci Rep. 2022 May 5;12:7350. doi: 10.1038/s41598-022-11153-4 (PMC9072334; doi:10.1038/s41598-022-11153-4)
Supplement: Supplementary file 1 — Supplementary Information. [file 41598_2022_11153_MOESM1_ESM.pdf]

Spatial maps and oscillations in the healthy hippocampus of Octodon degus, a natural model of sporadic Alzheimer's disease

Matias Mugnaini[1], Diana Polania[2] , Yannina Diaz[1], Marcelo Ezquer[3], Fernando Ezquer[3], Robert M. J. Deacon[2], Patricia Cogram[2,4]* and Emilio Kropff [1]*

*Joint senior author
[1] Leloir Institute – IIBBA, CONICET, Buenos Aires, Argentina.
[2] Institute of Ecology and Biodiversity, Department of Ecological Sciences, Faculty of Sciences, Universidad de Chile, Santiago, Chile
[3] Centro de Medicina Regenerativa, Facultad de Medicina, Clínica Alemana-Universidad del Desarrollo, Santiago, Chile.
[4] The Center for Neural Circuit Mapping, University of California, Irvine, Irvine, CA 92697.

Supplementary figures

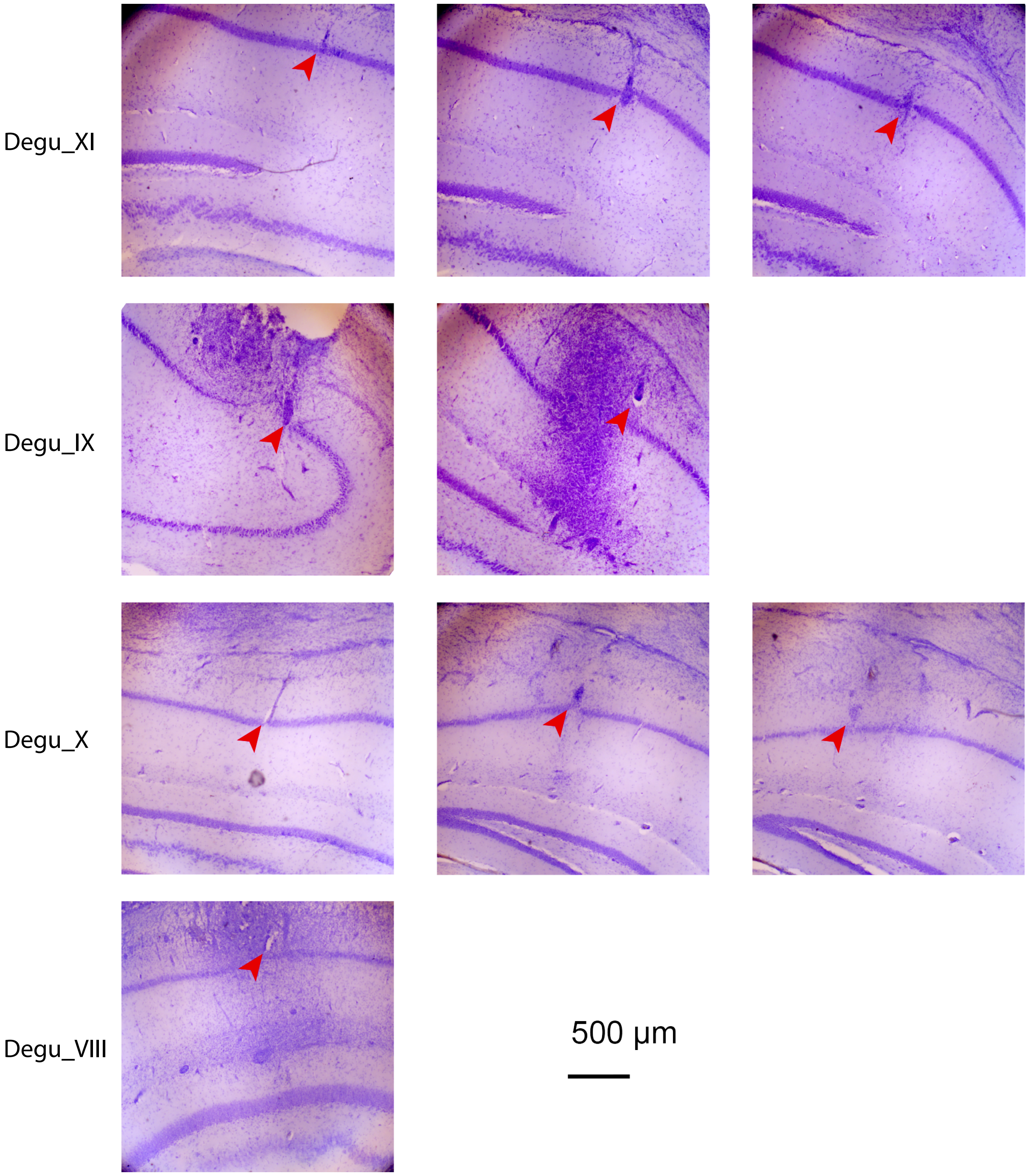

Figure S1 – Histology of recording sites in other tetrodes and animals. Cresyl violet stained histology of degus VIII to XI (one per row; tetrodes of degu XII are shown in Fig. 8). Each panel shows the slice where the tip of a CA1 tetrode was located (red arrow).

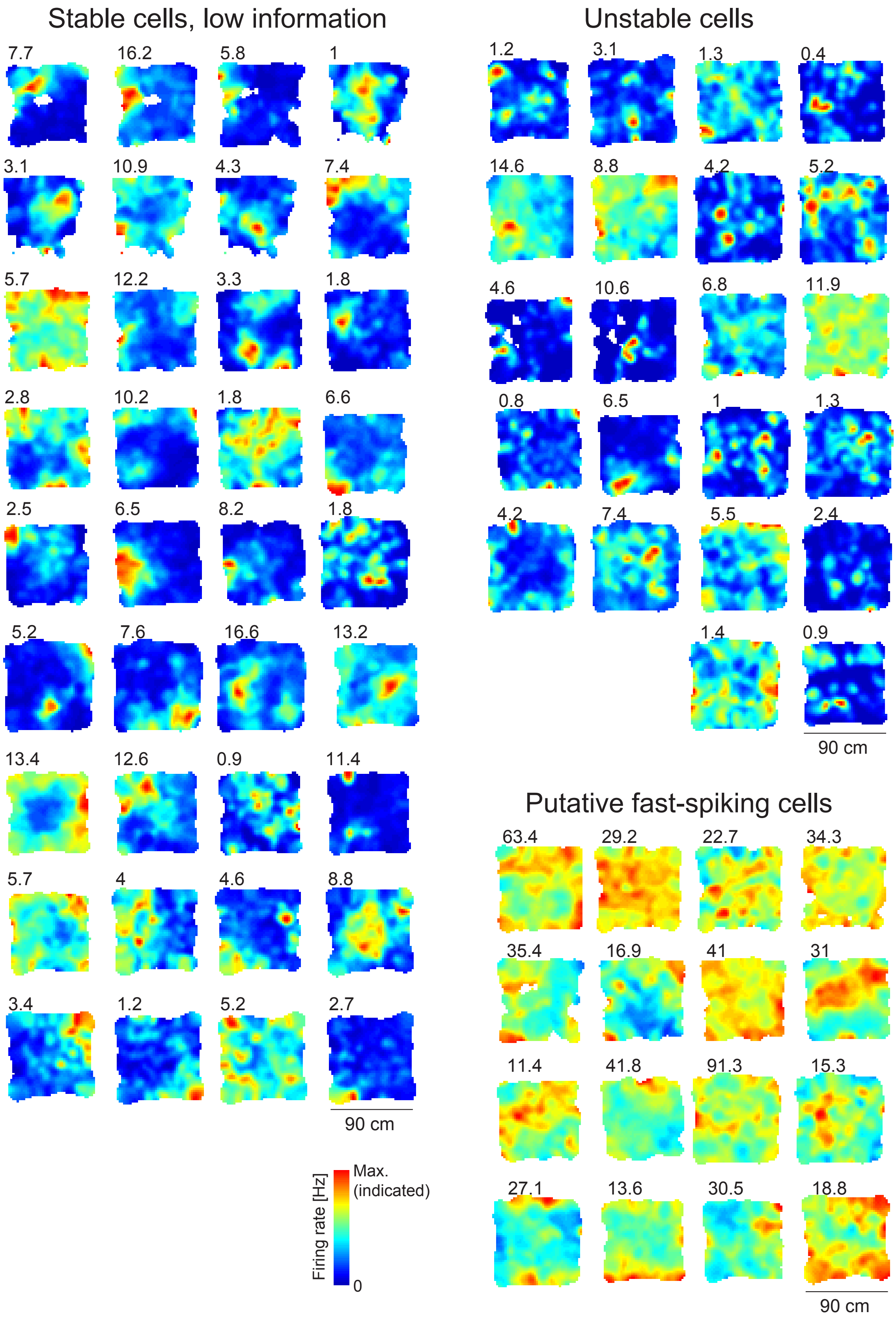

Figure S2 – Other recorded cell groups. Open field maps of stable cells with low information content, unstable cells and putative fast spiking cells, as indicated. Maximum firing rate in each map (corresponding to red) indicated.

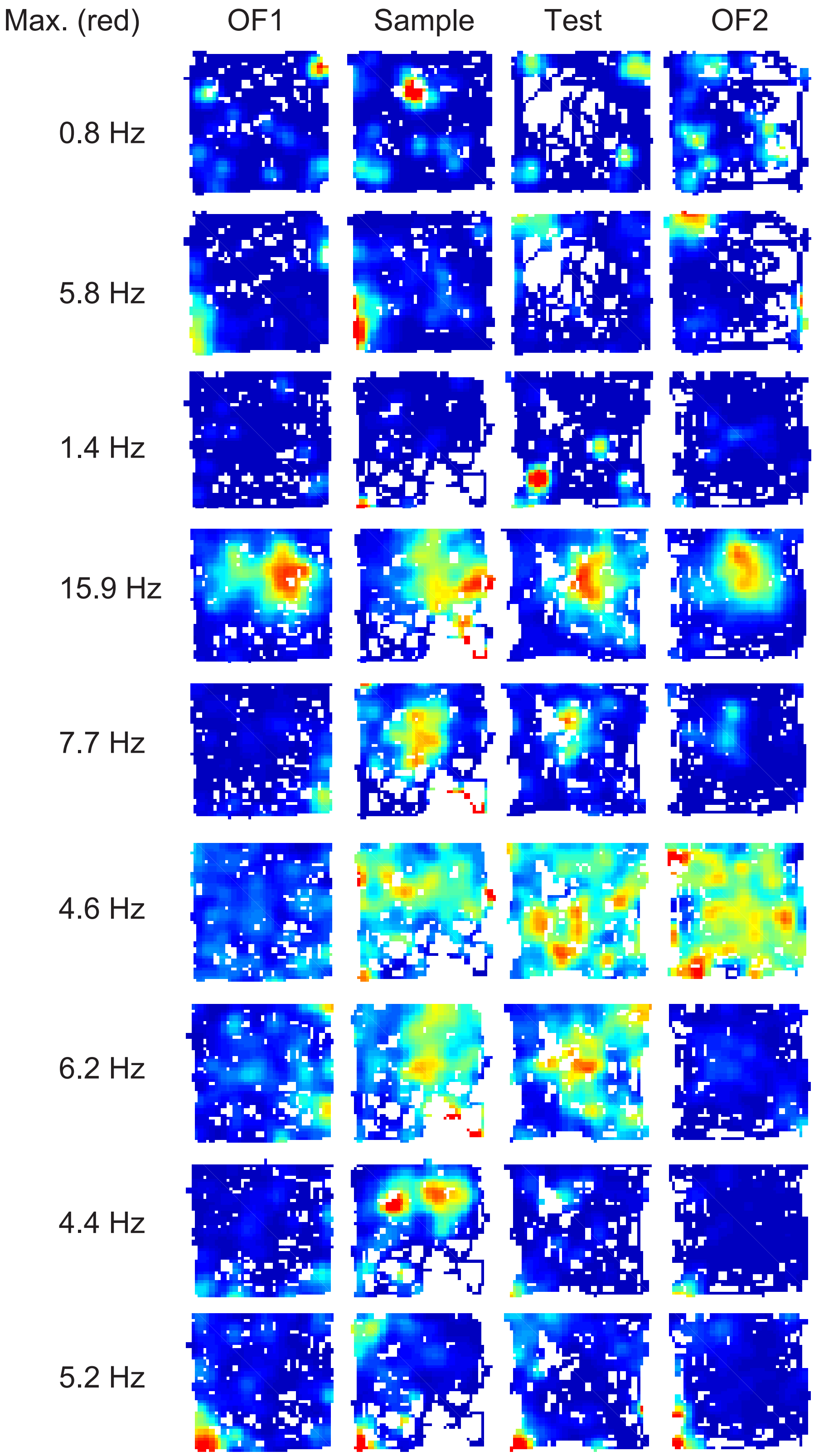

Figure S3 – Spatial maps during the objects experiment on day 1 (part 1). Every row shows the map of a cell across sessions. The color code is consistent across columns, with blue corresponding to 0 Hz and red to the maximum value, indicated on the left.

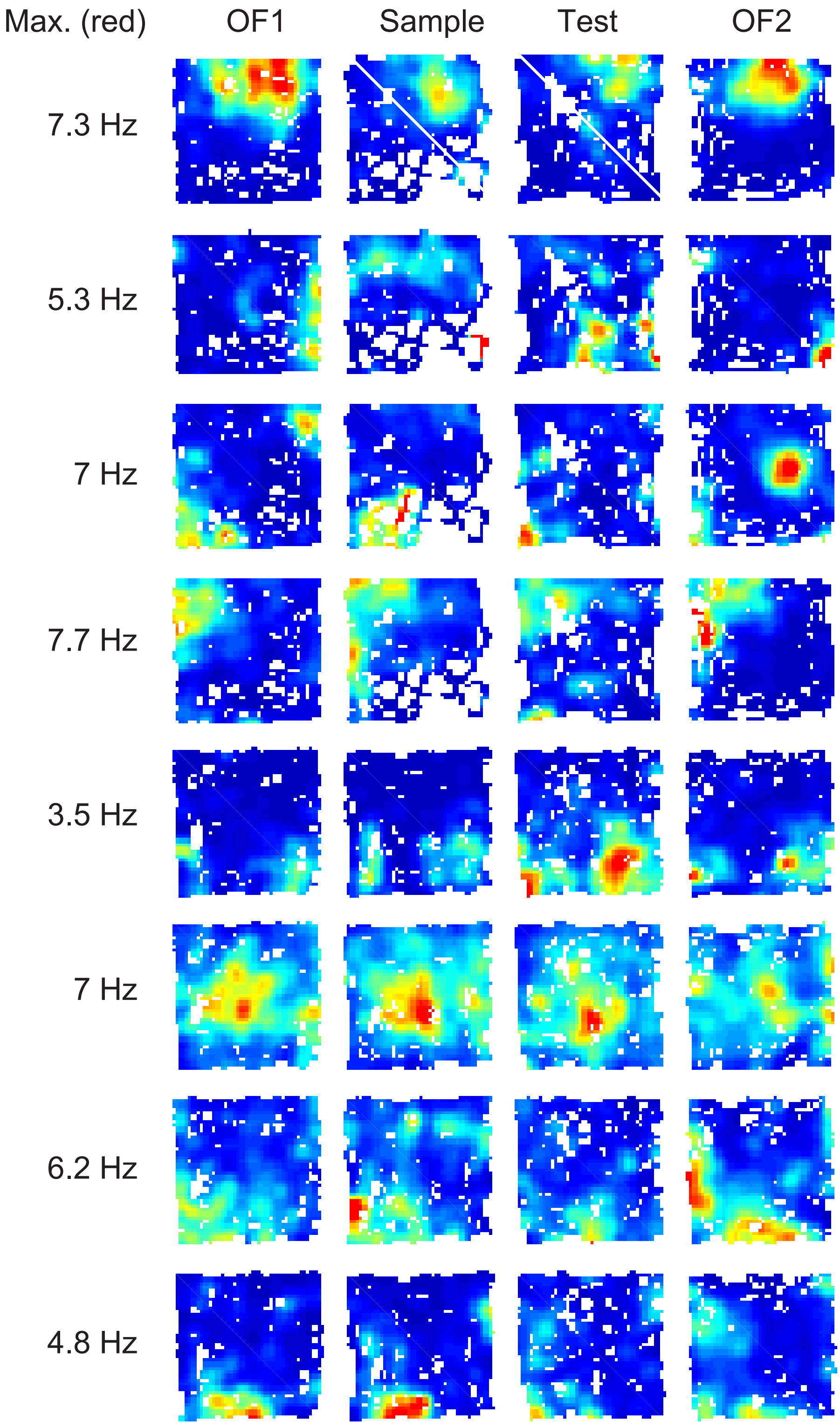

Figure S4 – Spatial maps during the objects experiment on day 1 (part 2). Every row shows the map of a cell across sessions. The color code is consistent across columns, with blue corresponding to 0 Hz and red to the maximum value, indicated on the left.
